# Non-Viral Targeted Nucleic Acid Delivery: Apply Sequences for Optimization

**DOI:** 10.3390/pharmaceutics12090888

**Published:** 2020-09-18

**Authors:** Yanfang Wang, Ernst Wagner

**Affiliations:** Pharmaceutical Biotechnology, Center for System-based Drug Research, Center for NanoScience (CeNS), Ludwig-Maximilians-Universität, D-81377 Munich, Germany; yangfang.wang@cup.uni-muenchen.de

**Keywords:** DNA-barcode, nucleic acid delivery, non-viral, peptide, pharmacological barriers, sequence-defined, supramolecular self-assembly, tumor-targeted

## Abstract

In nature, genomes have been optimized by the evolution of their nucleic acid sequences. The design of peptide-like carriers as synthetic sequences provides a strategy for optimizing multifunctional targeted nucleic acid delivery in an iterative process. The optimization of sequence-defined nanocarriers differs for different nucleic acid cargos as well as their specific applications. Supramolecular self-assembly enriched the development of a virus-inspired non-viral nucleic acid delivery system. Incorporation of DNA barcodes presents a complementary approach of applying sequences for nanocarrier optimization. This strategy may greatly help to identify nucleic acid carriers that can overcome pharmacological barriers and facilitate targeted delivery in vivo. Barcode sequences enable simultaneous evaluation of multiple nucleic acid nanocarriers in a single test organism for in vivo biodistribution as well as in vivo bioactivity.

## 1. Introduction

Nucleic acids, as plasmid DNA (pDNA), small interfering RNA (siRNA), micro RNA (miRNA), messenger RNA (mRNA), antisense oligonucleotides (ASOs) and clustered regularly interspaced short palindromic repeat (CRISPR)-associated nuclease 9 (Cas9)/single guide RNA (sgRNA) ribonucleoprotein (RNP) system have been used as therapeutic macromolecules for the treatment of severe and life-threatening diseases at the genomic level, such as for cancer or monogenetic defects [1,2,3,4,5,6,7,8,9]. Over the past few years, the number of human clinical trials for gene therapy has greatly increased [10]. More and more gene therapy-based products have been approved by the U.S. Food and Drug Administration (FDA), European Medicines Agency (EMA) and the China Medical Products Administration (NMPA, formerly China Food and Drug Administration-CFDA).

As compared with drugs of low molecular weight, therapeutic delivery of nucleic acids is far more complicated [11]. First, therapeutic nucleic acids are mostly negatively charged macromolecules, which prevents direct diffusion across the cellular lipid membranes. Second, naked nucleic acids are unstable in the bloodstream and will be rapidly degraded by nucleases and cleared by kidney after intravenously administration [12]. Appropriate delivery systems shall be used to stabilize and protect the nucleic acids to realize efficient cargo accumulation in targeted sites. Once internalized into the target cells commonly by the process of endocytosis, delivery systems shall promote escape from the endosome into the cytosol where they have to release their nucleic acid cargoes in bioactive form. Finally, nucleic acids need to reach their specific subcellular sites for therapeutic intervention. Different macromolecular cargos act at different intracellular target sites (Table 1); pDNA and Cas9/sgRNA need to function in the cell nucleus, whereas siRNA and mRNA act in the cytosol. This may require carriers with different functional domains for effective delivery. In addition, carriers are required to alter their biophysical properties in a dynamic mode (pH-, enzyme-, or redox-sensitive) during different phases of extra- and intracellular delivery [13]. Furthermore, different treatment modes (systemic, regional and local) as well as different target organs and cell types have different requirements for carriers.

Optimized by natural selection and evolution, viruses with diverse functional microdomains are the most potent and efficient carriers for nucleic acids. Various virus-based delivery systems have been developed and have been applied in more than half of the clinical gene therapy trials worldwide in the past few decades [10]. Despite their demonstrated high transfection efficiency, their immunogenicity, limited cargo-loading capacity, restricted tissue tropism and expensive production limit the application. Therefore, non-viral synthetic delivery systems have been developed as alternative [14]. Such synthetic carriers can be virus-inspired and contain different domains to mimic the efficient, dynamic virus-based infection process [15].

A series of different strategies are followed for synthetic nucleic acid delivery systems, including lipid-based, polymer-based or other nanomaterial-based formulations [12,16,17,18,19,20,21,22,23,24]. As outlined in the following section, a significant variety of sequence-defined artificial polymers was generated, with the aim to improve nucleic acid delivery and their bioactivity. Functional building blocks such as shielding domains, targeting ligands, hydrophobic domain and endosomal buffering agents can be incorporated to overcome the extra- and intracellular pharmacological barriers for better systemic application.

## 2. Sequence-Defined Macromolecular Carriers

Like natural viruses, non-viral biomimetic nucleic acid delivery systems shall be responsive to the changing biological environment by introducing different functional subunits. All design elements including the macromolecule size and overall charge, number, sequence and topology (linear, branched, comb, hyperbranched or dendritic structure, and attachment sites for additional functional groups) of subunits play a crucial role in the final transfer efficiency. The precise synthesis of such complex multifunctional macromolecules as defined sequences is essential, not only for pharmaceutical reasons of reproducible production, but also for identification of clear relations between the chemical structure and biological activity.

With the progress of macromolecular chemistry, precise sequence-defined artificial polymers can be generated and optimized to meet the requirement for specific delivery [25,26,27]. On the one hand, improved technologies such as reversible addition-fragmentation chain transfer (RAFT) [28] enable synthesis of multi-block copolymers with higher precision, enabling the evaluation of carriers with different numbers and sizes of bioactive domains [29,30,31]. This encouraging polymer direction is an important field on its own and goes beyond the scope of the current review. On the other hand, macromolecules can be assembled in a precise sequence by solid-phase-supported synthesis (SPS).

As a focus of the current section, sequence-defined peptide carriers and peptide-like artificial macromolecular structures can be designed benefiting from the progress of SPS. In the design process, artificial functional units for the specific delivery process based on artificial amino acids and lipids have to be identified. Then these multiple components are step-by-step assembled into macromolecules with a defined-sequence, and their nucleic acid delivery ability is evaluated. Afterwards, further improvements are made to optimize the delivery systems on the basis of the structure-activity relations. Initial pioneering work identified the required number of cationic amino acids as lysine, arginine or ornithine in precise defined macromolecular structures [32]. The incorporation of cysteines improved the polyplexes stability by disulfide cross-linking, and the incorporation of endosomal-buffering histidine [33] or membrane-destabilizing peptides [34,35] enhanced nucleic acid delivery efficiency by improving their endosomal escape capacity. 

### 2.1. Lysine-Based Sequence-Defined Peptides

Lysine has drawn significant interest for the design of sequence-defined peptides as carriers for nucleic acids due to its cationic nature and the ability to provide multiple coupling sites for different topologies [36]. It was found that a minimum of 6-8 cationic amino acids is required to compact pDNA into polyplexes active in gene delivery [32,37]. The DNA binding and compaction ability increases with the number of cationic groups [32]. The introduction of cysteines into poly-lysine peptides increased pDNA binding through disulfide cross-linking [38], enabling the formation of stable polyplexes by shorter peptides consisting of only six cationic lysines [39]. Meanwhile, the cross-linked polyplexes exhibited significantly higher gene expression in vitro as compared to the uncross-linked polyplexes, with peptides containing two cysteines mediating maximal gene expression [38]. Incorporation of histidine for enhanced endosomal buffering further improved the in vitro gene expression in the absence of lysosomatropic chloroquine [39].

Direct comparison of linear, dendritic and hyperbranched poly(*L*)lysine (PLL) found that branching was beneficial for transfection, yet displayed increased cytotoxicity as well as reduced enzymatic biodegradability [40]. PLL dendrimer combined the easily biodegradation properties of linear PLL with high transfection efficiency of branched PLL. The transfection efficiency was largely related with the generations, with the sixth generation (KG6) exhibiting high level of pDNA transfection efficiency in cells without significant cytotoxicity [36]. KG6/pDNA exhibited a longer blood circulation with more pDNA distribute in tumor tissues as compared with the DOTAP/Chol system in tumor bearing mice via intravenous administration [41]. In addition, PEGylated KG6 showed better systemic retention, lower organ distribution and effective accumulation in tumor sites [42,43]. By substituting the cationic terminal groups for arginines (R) to interact with the lipid-membrane and facilitate pDNA release, KGR6 exhibited a comparable pDNA compaction ability with significantly higher transfection efficiency than that of KG6 [44].

Mixson and coworkers [45,46,47,48,49,50] developed a series of linear and branched histidine-lysine (HK) peptides containing lysine (K) as the nucleic acid binding domains and histidine (H) as endosomal buffering domains for pDNA and siRNA delivery (Table 2). The optimized parameters are the ratio (e.g., H-K, H2-K and H3-K), degree of branching (e.g., linear, 4- or 8-branches), w/o histidine-enrich tails depended on nucleic acid type and the target cell lines [45,46,47]. Initial studies found that HK peptides with less than four branches were ineffective for nucleic acid delivery, unless used in combination with cationic carriers as liposomes [45,51]. The transfection efficiency was determined by the target cell types: a branched HK peptide was more effective than a linear HK peptide in transformed malignant cells [52], where the degree of branching was positively related with transfection efficiency; a linear HK peptide was superior to a branched HK peptide in primary cells [45], where the degree of branching was inversely related with transfection efficiency. The difference of the optimal HK peptides for different cells types was found to be closely correlated with the pH of endocytic vesicles, which was above 6.0 in transformed malignant cells and highly acidic with a pH of below 5.0 in primary cells [45]. By altering the percentage of cationizable histidines, the endosomal pH of a cell may determine the amount of nucleic acid released from the HK peptides. Addition of a histidine-enriched tail to a 4-branched H2K4b peptides with a repeating amino-acid sequence of -HHK- significantly increased the pDNA transfection efficiency [46]. While the effective 4-branched H2K4b peptides were ineffective for siRNA mediated gene silencing, 4-branched H3K4b peptides with the histidine-enriched repeat pattern of -HHHK- on the terminals exhibited more effectively augmented uptake of siRNA than H2K4b peptides [47]. Eight-branched H3K8b peptides with histidine-enriched domains (H8) and 8-terminal branches with the repeating -HHHK- sequence were found to be more effective as carriers of siRNA in comparison with H3K4b [47]. Application of HK peptides via intratumoral administration of Raf-1 siRNA to MDA-MB-435 tumor-bearing mice showed that siRNA nanoplexes formed by highly branched H3K8b were not particularly effective, yet the less branched H2K4b and H3K4b peptide were found to be the most effective carriers [48]. Meanwhile, 4-branched H3K(+H)4b peptide with an additional histidine for a better endosomal escape not only exhibited comparable gene silencing effect as compared with H3K8b for in vitro Raf-1 siRNA transfection, but also good antitumoral activity comparable with H3K4b for intravenous administration of Raf-1 siRNA nanoplexes to MDA-MB-435 tumor-bearing mice [53]. Further incorporating of H3K(+H)4b with cRGD as the target ligand via a polyethylene glycol (PEG) spacer triggered significantly greater gene silencing levels in targeted tumor tissues of MDA-MB-435 tumor-bearing mice via systemic administration [54]. Furthermore, the group found that in vitro screening often failed to identify the most effective candidates for in vivo. As compared with the proven effective pDNA delivery system formed by H2K4b, the ineffective linear H2K peptide was found to be far more effective in pDNA transfection to tumor xenografts in vivo via intravenous administration [49].

### 2.2. Cell-Penetrating Peptides (CPPs)

Cell-penetrating peptides (CPPs) are able to induce membrane translocation and/or endosomal escape for the delivery of nucleic acids into the cytosol or nucleus. Artificial pH sensitive and amphiphilic CPPs, like GALA, were developed to mimic the fusogenic properties of viral peptides [55]. GALA undergoes a conformation change in the acidic endosomal environment from random coil to an α-helical structure, which would facilitate endosomal escape and the subsequent nucleic acids release into the cell cytosol [56]. The anionic properties of GALA limited its application in nucleic acid delivery [57]. Cationic KALA and RALA peptides were designed by substitution of negatively charged Glu (E) in the GALA peptide sequence with positively charged Lys (K) or Arg (R) at a similar position on the sequence backbone, respectively (Table 3) [58]. KALA and RALA peptides maintained the fusogenic amphipathic characteristics of GALA, while achieving the ability to complex nucleic acids [58]. KALA peptide undergoes conformation change already at physical pH, which may disrupt other membranes and lead to toxicity. The most promising RALA peptide with selective endosomal disruption as well as reduced toxicity exhibited comparable transfection efficiency in pDNA [58,59], siRNA [60] and mRNA [61] delivery as compared with commercial lipofectamine or gold standard carrier systems.

By modifying a stearyl group at the *N*-terminus of transportan 10 (TP10), Langel and coworkers developed a new generation of chemical-modified CPPs, named the ‘PepFect’ and ‘NickFect’ families, for nucleic acids delivery (Table 3). The stearyl-TP10 (PepFect3) strongly enhanced the serum stability of complexes as compared to the unmodified CPPs [62], and exhibited comparable efficient pDNA transfer as commercial vectors both in vitro and in vivo [63]. By incorporating pH-titratable trifluoromethyl-quinoline analogues to the lysine-side chain of PepFect3 for the facilitating the endosomal release of cargos, the siRNA complexes of the designed PepFect6 significantly triggered the target gene knockdown via systemic administration without observable toxicity [64]. Instead of lysines and isoleucines in PepFect3, PepFect14 incorporated ornithines and leucines in the sequence [65]. By complexing with splice-correcting oligonucleotides (SCOs), the PepFect14/SCOs efficiently delivered SCOs into mdx mouse myotubes, a Duchenne’s muscular dystrophy (DMD) cell culture model, and induced splice-correction at rates higher than commercial vectors. For further improving the cellular internalization and endosomal escape, a phosphoryl-group was incorporated to the backbone of PepFect3 to obtain the NickFect1 with lower net charges, which exhibited a significant improvement of splice-correlation as compared with PepFect3 [66]. With ornithine as a side chain instead of lysine, NickFect51 exhibited promising endosomolytic properties [67] and greater transfection efficiency in nucleic acid delivery [68]. By altering the net charges and helicity, amphipathic α-helical peptide NickFect55 delivered pDNA into tumors in mice bearing intracranial glioblastoma or subcutaneous Neuro2a/HT1080 tumors [69].

### 2.3. Peptides with Supramolecular Self-Assembly Domains

The development of supramolecular self-assembly has enriched the field of nucleic acid delivery. Taking advantage of the natural propensity for hydrogen bonding within the secondary structure of peptides, the rationally designed sequence-defined peptides assemble into various nanostructures [73,74,75,76,77,78,79,80] via non-covalent interactions, such as hydrogen bonds, π–π interactions, electrostatic interactions and van der Waals interactions.

**Figure 1:**
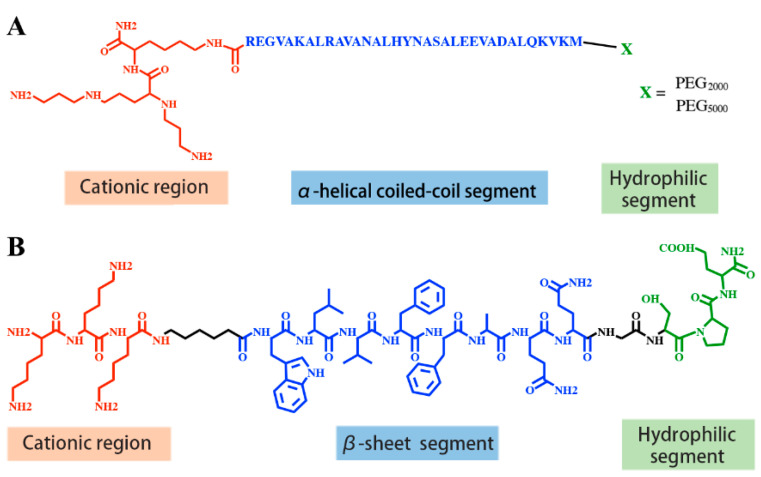
(**A**) Structure of peptides for virus-like nanoparticles formation. (**A**) Sp-CC-PEG, Reproduced with permission from [81], American Chemical Society, 2013. (**B**) K3C6SPD with the sequence of KKKC6WLVFFAQQGSPD. Reproduced with permission from [80], American Chemical Society, 2014.

Virus-like nanoparticles (VLPs) are one kind of structural viral mimicry derived from self-assembly of virus-derived peptides. Sequence-defined peptides were designed with a cationic region for nucleic acid compaction, an α-helical or β-sheet structure segment for supramolecular self-assembly and stability and a hydrophilic tail for nanoparticle dispersion. Lee and colleagues designed a short β-sheet peptide Glu-KW, with a repeat sequence of WKWE to promote β-sheet formation, a cationic K8 segment for nucleic acid binding and a carbohydrate d-glucose at the outermost part to prevent uncontrollable aggregation (Table 4). Filamentous nanoribbons assembled by Glu-KW and siRNA exhibited significant siRNA-mediated gene silencing [79]. Stupp and colleagues designed an α-helical PEGylated coiled coil peptide modified by cationic spermine segments (Figure 1A) [81]. The peptide was preassembled into mushroom aggregates, then dsRNA was encapsulated via electrostatic interactions to form filamentous virus-like nanoparticles [81]. Vries and colleagues designed a minimal viral coat polypeptide C-(GAGAGAGQ)_10_-K_12_, with an *N*-terminal oligolysine block for nucleic acid binding, a central silk-like sequence (GAGAGAGQ)_10_ for self-assembling into stiff filamentous structure and a C-terminal 407-amino acid hydrophilic random coil for dispersion (Table 4) [82]. The designed peptide co-assembled with pDNA [82] or mRNA [83] into rod-shaped VLPs, which protect the nucleic acids against enzymatic degradation and show significant transfection efficiency in cells. Chau and colleagues developed spherical virus-like nanococoons through coassembling pDNA and a short peptide K3C6SPD [80], which was consisted of an *N*-terminal cationic region for nucleic acid binding, a central β-sheet formation region for self-assembly and stabilization of the peptide/DNA nanococoons, and a C-terminal hydrophilic region for dispersion (Figure 1B). Further studies found that tuning the peptide sequence, such as changing side-chain hydrophobicity or β-sheet peptide length, significantly changes the stability of nanococoons, thus affecting their DNA transfection efficiency [84]. By incorporating four histidines at the *N*-terminus and two histidines within the central β-sheet region as pH-sensitive regions, as well as two aromatic benzylated cysteines CBzl within the central β-sheet region to promote self-assembly and inter-subunit association through π–π stacking and hydrophobic interactions, Chau et al. further designed H4K5-HC_Bzl_C_Bzl_H peptide, which coassembled with pDNA into spherical VLPs [85]. These VLPs displayed stimuli-responsive sequential disassembly and effective DNA transfection efficiency [85].

In addition, Stupp’s team developed a supramolecular self-assembly system based on peptide amphiphiles (PAs, Table 4), which consist of a short β-sheet forming peptide sequence linked to a hydrophobic tail [74,75,76,77]. DNA-PA nanofibers formed by DNA-PA conjugates self-assembly displayed improved binding affinity to the target protein, enhanced nuclease-resistance as well as improved capacity to block PDGF-BB activity as compared with free aptamer [86]. Hernandez-Garcia et al. synthesized P4 peptide by fusing P2 peptide for specific siRNA binding [87] and P3 peptide for structural switching from α-helix to β-sheet [88] via a spacer of two glycines [89]. siRNA-peptide nanoparticles formed with P4 via supramolecular assembly and exhibited efficient protein knockdown in glial neuronal cells without significant toxicity. This demonstrates the potential of using supramolecular systems for non-viral nucleic acid delivery [89]. Further discoveries on the important function offered by these self-assembling supramolecular nanostructures and the formed three-dimensional scaffolds will benefit the development of smart nucleic acid nanomedicines.

### 2.4. Lipo-Peptides

**Figure 2:**
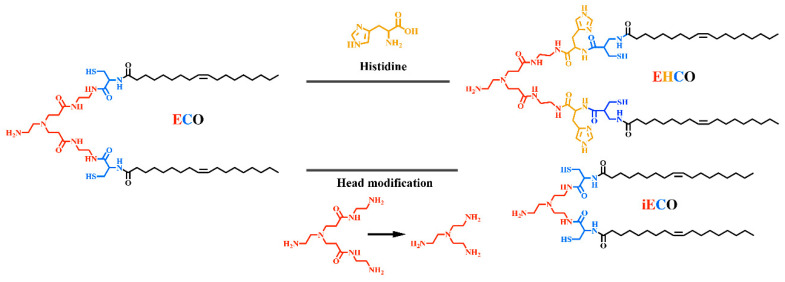
Chemical structure of ECO, EHCO and isotypic ECO. E: Ethylenediamine; C: Cysteine; O: Oleic acid; H: Histidines.

Besides using only natural amino acids, defined artificial lipo-peptides were also synthesized as nucleic acid carriers. Lu and colleagues [90,91,92] designed lipo-peptide like carriers for nucleic acid delivery, which consisted of a cationizable triethylene tetramine head, amino acid-based linkers including cysteines, histidines or lysines and two terminal hydrophobic oleic acids (Figure 2). Libraries were screened to optimize the number of protonable amines, the presence of histidines, and the unsaturated degree of fatty acid tails. Sequence-defined (1-aminoethyl) imino bis [*N*-(oleoyl cysteinyl histinyl-1-aminoethyl) propionamide] (EHCO) and (1-aminoethyl)iminobis[*N*-(oleicylcysteinyl-1-amino-ethyl)propionamide] (ECO) displayed high cellular internalization as well as reporter gene silencing upon the intracellular delivery of nucleic acids [93]. By incorporating retinylamine (Ret) to the ECO/pDNA nanoparticles via a PEG spacer for targeted delivery of pDNA into retinal pigmented epithelium (RPE), the resulting Ret-targeted ECO/pDNA nanoparticles significantly improved the electroretinographic activity of Rpe65−/− mice. The therapeutics effects lasted for at least 120 days [94]. The therapeutic potential of ECO/pDNA nanoparticles in the treatment of visual dystrophies was also confirmed by further research [95,96]. Malamas et al. [90] optimized the amphiphilic cationic lipid carriers by evaluation of the role of protonatable amine numbers and pKa of the cationic head group, the degree of unsaturation of the bis-hydrophobic tails and the presence of histidine residues as an amino acid linker. The amphiphilic cationic lipids displayed structure-dependent RNAi activity, with ECO (bis-oleic acid) and ECLn (bis-linolic acid) performing the best reporter gene knockdown. Later an ECO-based siRNA nanoformulation was applied for tumor-targeting therapeutics by incorporating an RGD peptide via a PEG spacer. Intravenous injections of RGD-targeted ECO/siβ3 nanoparticles significantly inhibited primary tumor growth and metastasis in MDA-MB-231 breast tumors bearing mice [97]. RGD-targeted ECO/siDANCR nanoparticles resulted in gene silencing of onco-lncRNAs and robust suppression of tumor progression without overt toxic side-effects in nude mice bearing triple-negative breast cancer (TNBC) xenografts [98].

### 2.5. Oligoaminoamines (OAAs)

**Figure 3:**
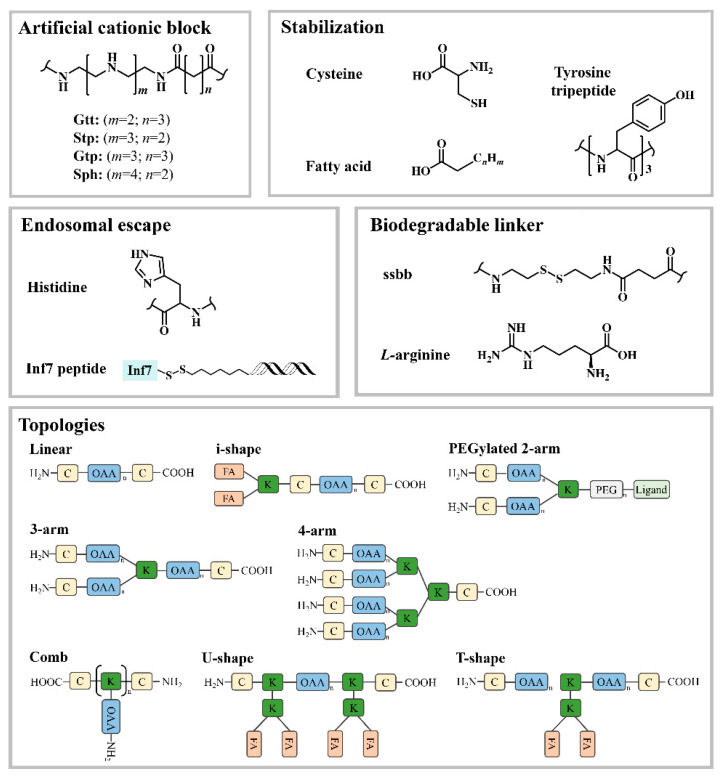
Functional elements for sequence-defined OAAs different topologies. ssbb: Disulfide building block = succinoyl-cystamine; OAA, artificial oligoamino acid; K: Lysine; C: Cysteine; FA: Fatty acid.

Hartmann and Börner adopted SPS for the synthesis of sequence-defined oligoaminoamines (OAAs) by using completely artificial building blocks, such as protected spermine and succinic acid. Disulfide linkages or terminal PEG chains were optionally introduced at precise positions. The resulting oligomers were firstly applied for pDNA polyplexes formation [27,99,100,101]. Extending this strategy, Schaffert et al. [102,103] developed novel artificial oligo(aminoethylene) amino acids with appropriate internal t-butyloxycarbonyl (tBoc) and amino-terminal fluorenyl-methoxycarbonyl (Fmoc) protective groups. The artificial oligoamino acids (tetraethylenepentamine artificial peptide Stp, Gtp and Ptp, pentaethylene hexamine peptide Sph, triethylenetetramine artificial peptide Gtt) contain several repeats of protonatable amino ethylene motif, which mediates the proton sponge effect in the gold standard transfection agent polyethylenimine (PEI) [104,105]. By introducing lysines as branching sites and cysteines as disulfide crosslinking for polyplexes stabilization, the resulting artificial oligoamino acids were applied to establish more than 1400 sequence-defined oligoaminoamides with different sequence and architectures (linear [106,107], 2-arm [107], 3-arm [107,108], 4-arm [102] [107,109], comb [110], i-, U- and T-shape [107,108,111]) for nucleic acid delivery. Hydrophobic domains as bis(acyl)-modified lysines, or tyrosine tripeptides were optionally incorporated for hydrophobic polyplex stabilization, and histidines for endosomal buffering (Figure 3).

Due to the precision of the chemical design, it is feasible to obtain clear structure-activity relations via sequence-defined oligopeptides. Testing the length of a linear oligo(aminoethylene) amides based on Stp (containing 3-protonable nitrogens per unit) demonstrated that 30 Stp units (representing 90 protonable nitrogens) was optimum for DNA compaction, with 6-fold higher pDNA transfection efficiency and 10-fold lower cytotoxicity than the conventionally “gold standard” linear PEI (LPEI, 22 kDa) [106]. Salcher et al. [102] developed 4-arm oligomers with 2 to 5 repeats of artificial oligoamino acid building blocks, and demonstrated that the introduction of *N*-terminal cysteines increased the stability and transfection efficiency of pDNA polyplexes. Furthermore, the building blocks exhibited a clear rank in the order of Sph > Stp >> Gtt in terms of pDNA compaction and transfection capability. Further optimization via fine-tuning the endosomal protonation capacity [109] found that building blocks with even numbered protonable nitrogens (Sph and Gtt) exhibited significantly higher endosomal buffer capacity than structures with an odd number (Stp) analog. Yet Gtt-containing oligomers (lowest number of nitrogens) exhibited low gene transfection efficiency due to less pDNA binding capacity. Incorporation of cysteines for stabilizing disulfide crosslinking compensated for transfection efficiency. This indicates that efficient pDNA delivery needs to be combined and balanced with both buffering and stabilizing moieties. Among sequence-defined 4-arm oligomers developed by Salcher et al. [102], Stp- and Sph-based cysteine-ended oligomers exhibited similar siRNA compaction capacity, with Stp-based oligomers providing the best reporter gene silencing. 

In addition to *N*-terminal cysteines for bioreducible stabilization, hydrophobic fatty acids were incorporated to control the hydrophobic polyplex stabilization and pH-specific lytic activity using lysine amines as the attachment site [107,111,112]. Lipo-oligomers with lysine diacylated with unsaturated C18 (oleic acids or linolic acids) mediated higher polyplex stabilization, higher lytic activity and higher transfection efficiency as compared with analogues without fatty acids or with a single fatty acid as well as with short length carbon chain fatty acids. Moreover, pH-specific lytic activity was controlled by the type of fatty acids used, while the position of the hydrophobic elements, as at the end/center/both ends of the backbone for the i-/T-/U-shape, respectively, had no significant influence. Incorporation of hydrophobic tyrosine tripeptides (Y_3_) [112,113] further increased the endosomal buffer capacity and serum stability via aromatic π–π stacking, resulting in enhanced pDNA polyplexes compaction as well as pDNA transfection efficiency as compared with the corresponding Y_3_-free oligomers.

For pDNA of larger size (5–15 kbp), it is crucial to compact it into small-sized nanoparticles by polycations and then release it in the nucleus in the bio-active form. 

Incorporating c-Met-binding peptide (cMBP2) as a targeting ligand into sequence-defined 2-arm or 4-arm architectures via a precise PEG shielding domain exhibited far higher pDNA transfection efficiency in cell culture than the corresponding non-targeted groups [114,115]. Meanwhile, incorporation of alternating histidines into the cationic core for enhanced endosomal buffer capacity was found to be strongly beneficial for pDNA transfection [114,115]. However, pDNA polyplexes were not perfectly compacted by the PEG-containing carriers, resulting in a poor performance in the in vivo situation after intravenous administration. Therefore, co-formulations of a multifunctional cMBP2-containing 2-arm structure with an analogous PEG-free, well compacting 3-arm structure exhibited greatly enhanced in vivo gene expression in Huh7 tumor bearing mice [115]. This discrepancy between pDNA compaction and nanoparticle shielding was studied in detail by screening of two-arm oligoaminoamides containing 37 cationizable nitrogens and hydrophilic segments of different lengths: either PEG segments with 12, 24 or 48 oxyethylene repeats or peptidic shielding blocks composed of 4 or 8 repeats of the proline-alanine-serine sequence [116]. Interestingly, only the shorter hydrophilic segments (12 oxyethylene units or four PAS repeats) resulted in very compact 40–50 nm pDNA polyplexes, similar as 3-arm structures without a hydrophilic segment. Obviously, consistent with other reports [117,118,119,120], the balance between shielding, pDNA compaction and sufficient endosomal buffering must be considered for optimizing non-viral carriers based on hydrophilic and cationic block oligomers.

For siRNA delivery, the introduction of fatty acids (unsaturated oleic acids or linolic acids) was found especially beneficial for hydrophobic stabilization and pH-specific lytic activity. Incorporation of tyrosine tripeptides for polyplex stabilization by aromatic π–π interactions further enhanced siRNA delivery. The presence of cysteines was strictly required for siRNA polyplexes stabilization and gene silencing efficiency for the majority of structures. The stability provided by cysteine disulfide crosslinking can be partly compensated in U-shape or T-shape structures by extra hydrophobic stabilization devoted by the fatty acids.

For better mimicking the behavior of viruses in a dynamic and bioresponsive way, stimuli-responsive domains were introduced to a specific site of sequence-defined OAAs via SPS, which would mediate cleavage of chemical bonds or changes of characteristics responding to the biological microenvironment. Klein et al. [121] developed bio-degradable T-shape lipo-OAAs by introducing Fmoc-protected succinoyl-cystamine as the disulfide building block (ssbb) between cationic and lipid domains. The cytosolic glutathione (GSH) triggered favorable cytosolic siRNA release and degraded the lipo-oligomers into neutral lipids and nontoxic small hydrophilic elements. Reinhard et al. [122] precisely inserted short enzymatically cleavable (l)-arginine peptides (RR) between lipids and cationic domains of the T-shape lipo-OAAs. This resulted in endolysosomal protease cathepsin B-triggered siRNA release and reduced cytotoxicity.

## 3. Optimizing Carriers for Different Types of Therapeutic Nucleic Acids

On the one hand, as nucleic acids are mostly composed of an anionic phosphodiester backbone, their delivery requirements have similarities. On the other hand, the requirements differ for different nucleic acids due to their different physical properties and target sites as well as their specific applications. Therefore individual screening of nanocarriers for their optimized delivery is needed. pDNAs are large polymeric polyanions that require carriers for reversible compaction into nanostructures. Due to the much smaller size of siRNA and miRNA, their electrostatic interaction with cationic polymers is far less, resulting in limited polyplex stability. In terms of sgRNA with medium size, it is essential to interact with Cas9 protein properly for the subsequently genome editing function. For medium large sized mRNA, it is crucial to compact it into small-sized nanoparticles by polycations and then release it in the cytosol in the bioactive form. 

For the successful delivery of all cargos, the formation of stable nanoformulations with proper size, zeta potential, stability and suitable protection of cargo in the bioactive form is required. Nanoformulations need to realize endosomal escape and the cargos need to be available in the cytosol (siRNA, miRNA and mRNA) or be imported into the nucleus (pDNA and Cas9/sgRNA) for efficient genetic intervention.

### 3.1. Stable Nanoparticle Formation Is Important for siRNA Carriers

In 2018, the first siRNA-based therapeutic product, Onpattro (Patisriran), obtained the regulatory approval for the treatment of peripheral nerve disease caused by hereditary transthyretin-mediated amyloidosis (hATTR) [123]. Onpattro encapsulates siRNA against TTR mRNA into 60-100 nm PEGylated lipid nanoparticles (LNPs). After intravenous administration, the LNPs are coated with apolipoprotein in blood circulation, which facilitates LDL receptor targeted delivery into liver hepatocytes. Subsequently in 2019, the FDA also approved Givosiran (GIVLAARI), a completely chemically modified tri-(*N*-acetyl-galactosamine) (GalNAc)-PEG-siRNA conjugate as therapeutic for the treatment of patients with acute hepatic porphyria (AHP) [124]. The complete chemical stabilization of siRNA is required to avoid its biodegradation. Upon subcutaneous administration, the siRNA conjugate is selectively delivered into hepatocytes via asialoglycoprotein receptor (ASGPR) mediated endocytosis. These two FDA-approved siRNA therapeutics were important breakthroughs in the field of nucleic acid therapy beyond the previous established antisense oligonucleotide drugs [125]. They open the door of the medicinal market for a series of other rationally designed siRNA therapy products. High stability (by either liposomal encapsulation or covalent attachment of stabilized siRNA) and potent receptor-mediated delivery (by either targeting the LDL receptor or the ASGPR of hepatocytes) are important reasons for their efficacy. Future siRNA drugs targeting other tissues beyond the liver remain to be developed.

siRNA delivery systems share with pDNA delivery several critical steps such as endosomal escape or the subsequent cargo release. The functional elements that are beneficial for pDNA transfection may turn out to be also efficient constitutes for siRNA mediated gene silencing. As compared with pDNA, siRNA however is a far smaller double-stranded nucleic acid with 42-46 negative charges, which contributes much less to the electrostatic stabilization of polyplexes. Strategies such as covalent conjugation of siRNA with transport carriers, chemical optimization of the polycationic carriers with terminal cysteines, tyrosine tripeptides and hydrophobic fatty acids were adopted to enhance the extracellular stability of siRNA polyplexe (see Section 2). Meanwhile, moieties for polyplex stabilization shall be moderated and balanced with the endosomal release and the final transfection efficiency. Bioreducible disulfide crosslinking of terminal cysteines to stabilize polyplexes extracellularly was identified to be crucial for siRNA transfection efficiency in many studies. Yet further stabilization of polyplexes by cysteine-arginine-cysteine (CRC) motifs led to a reduction of gene silencing efficiency [126]. As also demonstrated in studies of Mixson’s lab, the optimal pDNA carrier H2K4bT [46] was less effective for siRNA delivery. In contrast, 8-branched H3K8b peptides containing more branches for siRNA binding were found to be more effective in siRNA mediated gene silencing than H2K4bT [47] (see Section 2). By adding a lysine to each terminal branch of H3K8b peptides, their siRNA binding ability further increased. However, this triggered less effective gene silencing [47].

The incorporation of endosomal buffering histidine, which was proven as beneficial for pDNA delivery, may reduce the stability of siRNA polyplexes. The loss of stability by histidine can be compensated by other stabilizing units. Luo et al. [127] demonstrated histidine-free T-shape lipo-oligomers with a short cationic Stp backbone (13 protonatable amines) exhibited better transfection efficiency than the corresponding histidine-containing oligomers. While for T-shape lipo-oligomer with a long cationic Stp backbone (25 protonatable amines), the incorporation of histidines further increased the siRNA transfection efficiency [128]. The finding is well consistent with previous reports. Histidine-enriched H3K8b peptides containing larger cationizable oligo-lysine exhibited significantly higher gene silencing efficiency than the corresponding histidine-free peptide [47]. Histidine-free siRNA carriers such as ECO and ECLn containing a short cationizable head group performed better gene silencing than their corresponding histidine-containing carriers [90].

### 3.2. Other Nucleic Acid Cargos Including Cas9/sgRNA or PMO

Precise genome modifying nucleases, such as CRISPR-associated nuclease Cas9 have been harnessed to create a DNA double-strand break (DSB) for site-specific genome editing [129,130,131,132]. Among different intracellular delivery of genome-editing agents in the form of gene expression constructs based on DNA [23] or mRNA [132] or Cas9 protein/sgRNA RNP formulations, Cas9/sg RNPs [23,131] were considered to be the most effective tool for the genome engineering duo to their immediately genome editing process without transcription or translation and risk of spontaneous genome integration. Intracellular delivery of the Cas9 protein across the cell membrane and escape from endosome in the form of active Cas9/sgRNA RNPs is a persistent challenge.

Lu and colleagues [133] replaced 3,3′-[(2-aminoethyl)imino]bis[*N*-(2-aminoethyl)propenamide in previously designed ECO by 2,2′,2″-triaminotriethylamine as a novel cationizable headgroup, and optionally introduced lysine or histidine as an additional linker. ECO and iECO (isotypic ECO without additional lysine or histidine) formed stable nanoformulation with pDNA, and mediated the most efficient GFP gene editing for the intracellular delivery of pDNA expressing CRISPR/Cas9.

Kuhn et al. [134] screened an analogous T-shape lipo-oligomer library containing saturated stearic acid, mono-/bis-unsaturated and amide functionalized or hydroxylated stearic acid lipid (OHSteA) moieties for Cas9/sgRNA RNPs delivery. Tyrosine tripeptides and terminal cysteines were adopted due to their beneficial effect in polyplex stabilization found in the context of pDNA and siRNA delivery. The Cas9/sgRNA lipo-complexes formed by cationic T-shape lipo-OAA T-OHSteA and negative Cas9/sgRNA were identified to be the best-performing formulations as compared to the ones formed by oligomers containing unsaturated or saturated stearic acid without hydroxylation. T-OHSteA Cas9/sgRNA complexes exhibited smaller and more defined particle formation, enhanced cellular internalization and improved endosome escape. This resulted in an increased nuclear association and the highest CRISPR/Cas9 mediated GFP gene knock-out efficiencies in Neuro2a eGFP-Luc cells and HeLa eGFP-Luc cells.

For the small noncharged cargo phosphorodiamidate morpholino oligomer (PMO), Lächelt and colleagues screened an analogous library of T-shape lipo-OAAs containing fatty acids with different numbers of unsaturated bonds. They found lipo-OAA/PMO conjugates containing linolenic acid with three cis double bonds exhibiting the highest splice-switching effect in vitro and in vivo after intratumoral administration [135]. The superior endosomal lytic activity of linolenic acid in lipo-OAA/PMO conjugates over corresponding saturated stearic acid or unsaturated fatty acids with other numbers of double bonds was further identified to be the most possible reason.

## 4. Pharmacological Barriers for In Vivo Delivery

**Figure 4:**
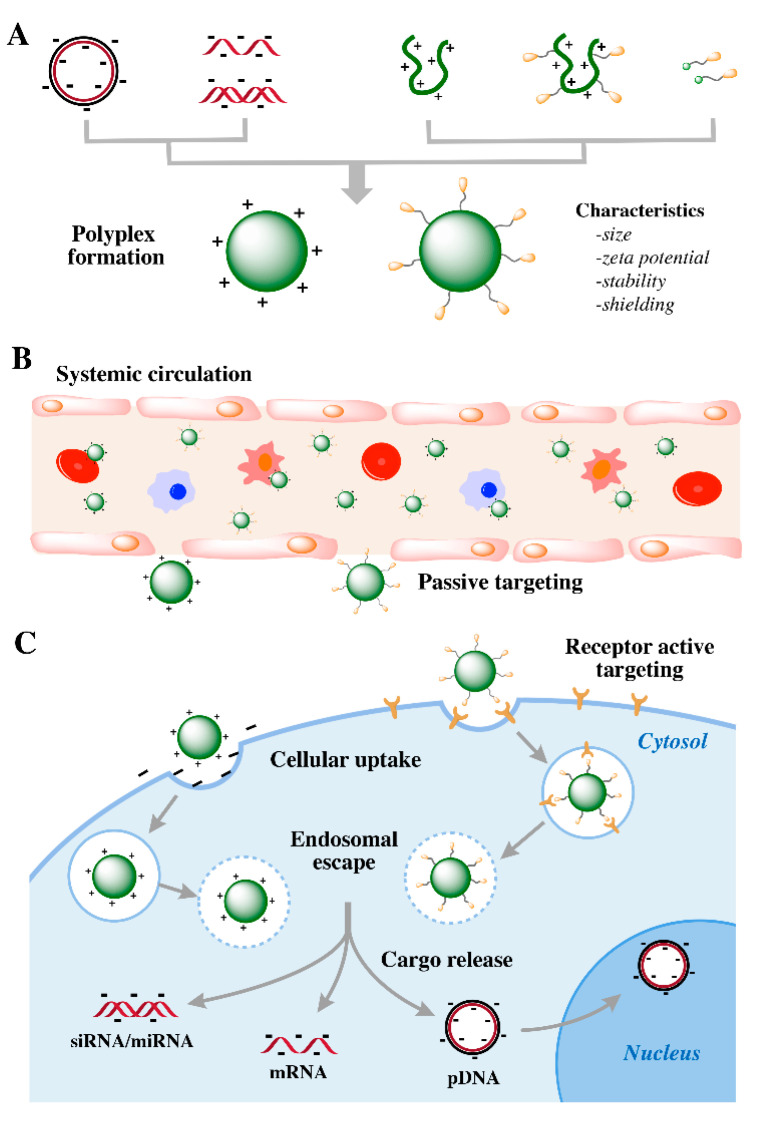
Pharmacological barriers for systemically administered non-viral targeted nucleic acid delivery. (**A**) Production of stable nucleic acids-loaded polyplexes. (**B**) After intravenous injection, nanocarriers shall avoid unspecific interactions with blood components and rapid clearance, accumulating in targeting areas during circulation. (**C**) After the passage of fenestrated blood vessels, nanocarriers shall be internalized by target cells via an active transport process, and realize efficient endosomal escape and release the cargos in an active form for gene expression or regulation.

Non-viral nucleic acid delivery systems have to overcome several extra- and intracellular pharmacological barriers to realize safe and efficient gene therapy (Figure 4). Nucleic acids shall be protected by nanomaterials from enzymatic degradation, non-specific interactions, rapid renal filtration and entrapment by phagocytes. Upon reaching the targeted sites, the nanoformulations need to overcome the vascular barrier and enter the target cells by cellular uptake. Subsequently they must escape from the endosome and release the nucleic acid in the bioactive form to trigger efficient gene regulation at the target site. The major pharmacological challenges and the corresponding strategies to overcome them are described as follows.

First, the carriers must form stable nanoparticles to prevent the naked nucleic acids from being degraded in the blood by nucleases and cleared from the bloodstream. As described in the above sections, the formation of nucleic acid complexes commonly is based on electrostatic interactions between anionic nucleic acids and cationic domains of the sequence-defined polymers [47,90,102]. Additional stabilization can be donated by hydrophobic interactions by incorporated lipids [90,108], aromatic stabilization by tyrosine tripeptides [112], bioreducible disulfide crosslinking formed by cysteines [90,102] or α-helical or β-sheet structure segments for supermolecular self-assembly [79,80,81,82,83,84,85].

After intravenous administration, polyplexes interact with electrolytes, proteins or non-target cell in the serum, which might result in the dissociation of polyplexes and a loss of gene transfection. Moreover, innate immune responses caused by the binding of positive charged polyplexes with serum complement proteins, self-aggregation into larger microstructures and aggregation with erythrocytes and other blood cells would trigger adverse effects. Shielding the polyplexes with PEG, a carbohydrate or other hydrophilic polymers must reduce such undesired non-specific interactions with the bioenvironment; such measures also should prolong the circulation time in the blood stream [54,117,136,137,138]. However, PEGylation of positive charged surface reduced cell association and the following endosomal escape function of the aminoethylene-based OAA polyplexes, which was consistent with previously finding with PEGylated PEI polyplexes [118,139]. This dilemma can at least be partly compensated by incorporating target ligands for receptor-mediated internalization and histidines for enhanced endosomal buffer capacity.

Afterwards, upon proper surface shielding and thus better biodistribution, the carriers shall accumulate at specific target tissue sites. The hydrodynamic size of polyplexes plays a critical role on the biodistribution and pharmacokinetics for systemic administration. Nanoparticles with a hydrodynamic size around or below 6 nm are rapidly cleared from the blood stream by the kidney [140]. In tumor-bearing mice, larger polymers or long-term circulating nanoparticles of sizes between 20 and 400 nm can leave the blood circulation across a leaky tumor vasculature and passively accumulate in the interstitial space of tumor tissues, as described by the enhanced permeation and retention (EPR) effect [141,142]. Once accumulated at tumor sites, specific binding with target tumor cells and subsequent effective cellular internalization can be facilitated by incorporated targeting functions. For example, targeting ligands such as B6 [109,143], cRGD [143], folic acid (FolA) [109,113], methotrexate (MTX) [144], c-Met-binding peptide (cMBP2) [114,115], transferrin (Tf), AP-1, EGF receptor-binding peptide (GE11) [143] and IL-6 receptor binding I6P7 peptide [145] were attached to precise PEG shielding domains and then introduced to the surface of polyplexes by pre- or post-modification for active receptor-mediated accumulation in target tumor cells.

**Figure 5:**
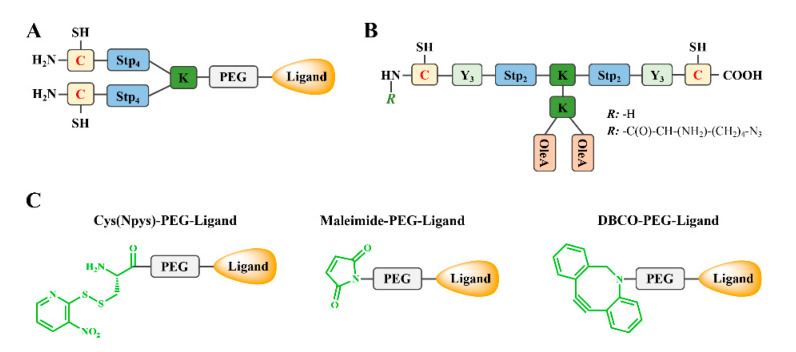
(**A**) Typical 2-arm ligand-PEG-OAA for all-in-one nano-formulations with shielding and targeting domains. (**B**) T-shape lipo-OAA with terminal cysteine or azide group for post-modification via disulfide-formation or orthogonal cooper-free click reaction, respectively. (**C**) PEG-ligands with specific attachment sites for post-modification.

In case of the all-in-one formulations with targeting and shielding domains (Figure 5A), pDNA polyplexes formed by 2-arm/4-arm ligand-PEG-OAAs exhibited an average size of ~100 nm, and the histidine-incorporation in the backbone significantly improved the pDNA transfection efficiency as compared to those control groups with alanine in the backbone, or than to the corresponding non-targeted groups [140,146,147]. After intravenous injection of I6P7-PEG-Stp-histidine/pDNA polyplexes developed by Huang et al. [145], the delivered pING4 (pDNA encoding inhibitor of growth 4) was found successfully expressed in the glioma, resulting in a significantly prolonged survival time of treated orthotopic U87-bearing mice. As for much smaller siRNA, 2-arm/4-arm ligand-PEG-OAAs [140,146,147] formed multifunctional nanoplexes with an average size as small as ~8 nm. The resulting nanoplexes need to be stabilized by cysteines-based disulfide crosslinking, while siRNA needs to conjugate with an endosomolytic influenza peptide (Inf7) [148,149] to enhance the endosome escape capacity and subsequent gene silencing efficiency. Although siRNA nanoplexes were rapidly cleared by the kidney due to their small size, localized intratumoral administration exhibited superior tumor suppression and enhanced antitumoral activity via EG5 gene silencing [140,146]. To provide efficient alternatives for an all-in-one tumor targeted delivery system, non-shielding cationic OAAs were incorporated into ternary complexes to increase the average size of polyplexes to ~100 nm. Targeted combination polyplexes (TCPs) [150] were developed by coformulating siRNA with a 2-/4-arm FolA-PEG-OAAs and non-PEGylated 3-arm OAAs. DTNB (5,5′-dithio-bis(2-nitrobenzoic acid)) was reacted with one thiol-containing oligomer to activate the terminal cysteine thiol groups. The resulting TNB-modified OAAs reacted with the terminal cysteine groups of the other thiol-containing OAAs rapidly via disulfide formation. Later targeted lipo-polyplexes (TLPs) were developed [151] by first formulating siRNA with a T-shape lipo-OAA and then coformulating with a 2-arm FolA-PEG-OAAs via disulfide formation. Both the folate receptor (FR)-targeted TCPs and TLPs exhibited siRNA accumulation into a subcutaneous L1210 leukemia site and triggered a reduction of EG5 gene silencing via intravenous administration.

In addition to the all-in-one synthesis, the other option is to post-modify the cationic polyplexes core with a shielding and targeting shell at specific sites (Figure 5B,C), which allows the individual optimization of the core polyplexes and the shell ligands. Two strategies were developed for the post-modification of stable core siRNA lipo-polyplexes with targeting ligands. One was coupling cysteine ended oligomers formed NPs with maleimide- or ortho-pyridyl disulfide (OPSS) containing PEG-ligands via a cysteine-based linkage [152,153,154,155], the other was coupling with azido ended oligomers formed NPs with dibenzocyclooctyne amine (DBCO) PEG-ligands via an orthogonal cooper-free click reaction [127,156,157,158]. Nanoparticle shielding with PEG and related hydrophilic polymers results in surface charge masking, reduced unspecific biological interaction and enhanced systemic circulation [159]. However, shielding may interfere with subsequent cellular uptake and especially with endosomal escape. Therefore bioreversible attachment of shielding agents such as by endosomal labile linkers has been successfully explored [118]. Beckert et al. [160] designed 4-arm OAAs with additional lysine residues for subsequent post-modification with amine-reactive poly(*N*-(2-hydroxypropyl)methacrylamide) (pHPMA) by using the acid-labile linker AzMMMan. Upon intravenous administration in Neuro2A tumor bearing mice, such a bioreversible pHPMA shielding of pDNA polyplexes reduced unspecific expression in the first-pass organs such as the lung and the liver. Consistent with improved circulation and subsequent deshielding in endosomes, the shielding enhanced the gene expression in the distant tumor. Morys and colleagues post-modified core pDNA lipo-polyplexes with sequence-defined mono- or bivalent Cys(Npys)_2_-PEG_24_-GE11 via disulfide formation, the resulting post-functional lipo-polyplexes exhibited receptor-dependent internalization as well as luciferase marker gene and sodium iodide symporter (NIS) gene expression in epidermal growth factor receptor (EGFR)-overexpressing tumor cells. siRNA lipo-polyplexes modified with maleimide-gE4-FolA via disulfide formation showed FR-mediated internalization in vitro and extended persistence in L1210 tumor bearing mice [155]. Tf-conjugated siRNA lipo-polyplexes modified maleimide-PEG-Tf exhibited enhanced gene silencing in vitro and improved tumor persistence in murine Neuro2A tumor bearing mice [152].

As compared with the disulfide bonding reaction, click chemistry was highly specific and biorthogonal, without byproducts [161,162,163] or affecting the crosslinking of cysteines. FolA-conjugated siEG5 nanoparticles, designed by click-modification with DBCO-PEG-FolA, displayed extended tumor retention in L1210 tumor-bearing mice after intravenous application, with a knockdown of ~60% of target EG5 gene silencing [157]. Furthermore, EGFR-targeting lipo-polyplexes click-modified with GE11 were applied for EG5 siRNA/pretubulysin (PT) [156] co-delivery; significant combination effects were confirmed in EGFR positive tumor cell cultures. A novel dual antitumoral conjugate of EG5 siRNA with the pro-apoptotic peptide KLK (siEG5-KLK) was formulated into lipo-polyplexes, followed by DBCO-PEG-AP1 click modification for IL4 receptor mediated tumor targeting. The resulting nano-formulations showed an enhanced anti-tumor effect due to the combined effect of EG5 gene silencing induced mitotic arrest and KLK induced mitochondrial destabilization [127].

Targeting ligands as mentioned above recognize their specific receptors that are overexpressed in tumor cells with high metabolic activity and excessive proliferation, and then induce active receptor-mediated intracellular accumulation. Once accumulation of nanoparticles in endosomal vesicles of target tumor cells occurs, the upcoming intracellular hurdle is an effective endosomal escape. Sequence-defined polycations based on protonatable amino ethylene motif will promote its endosomal escape due to the proton sponge effect [164]. pH-responsive endosomolytic peptides, as Inf7 [148], can be introduced to further facilitate an endosomal escape. Influenza hemagglutinin HA2 derived peptides like Inf7 undergo conformational changes in the secondary structure in an acidic endosomal environment to an amphipathic helix. This leads to the de-stabilization of the endosomal membrane [148]. Hydrophobic domains, such as fatty acids, can be incorporated due to their endosomal pH-specific lytic activity. Amino acids as histidine, are optionally incorporated due to their endosomal buffering capacity in the acidic endosomal environment. 

The events following endosomal escape are still poorly understood. Studies have shown that only quite a few internalized siRNA could be delivered into cell cytosol, with a narrow window of siRNA released from endosomes within 5–15 min after cell internalization was observed [165,166]. It has also been reported that siRNA-mediated gene silencing ability was improved by siRNA complexes with continued endosomal escape (over hours) [151] rather than a short immediate endosomal escape.

In contrast to siRNA or mRNA, pDNA has to overcome the additional hurdle of nuclear entry, which has been proved to be a real bottleneck via cell cycle studies [167,168,169,170,171]. Nuclear import takes place during cell division in proliferating cells, and by a size-dependent active manner via the nuclear pore complex (NPC) in non-proliferating cells [172]. It was reported that coupling of DNA with a short cationic peptide as a nuclear localization signal (NLS) [172,173,174,175,176,177] could enhance pDNA transfection efficiency not only by improving nuclear entry [173,178] but also by facilitating cytoplasmic transport [178]. Other options such as incorporation of chromatin-targeting peptides [179], histones [180,181], phosphorylation responsible peptides [179] or microtubule-binding peptides [182,183] were evaluated for better nuclear import. Further research for improved intranuclear delivery is still highly requested.

Beyond an endosomal escape and nuclear entry barriers, nucleic acids should be released in the bioactive form via nanoparticle disassembly caused by disassembly of non-covalent interactions or physicochemical properties and conformation changes, or bond cleavages [13,122,126]. That means, polyplexes should be dynamic for nucleic acid delivery; they should be stable during the whole process of extra- and intra-cellular delivery to protect their loaded nucleic acids, but should disassemble in the cytosol and release the cargos at their target sites of action. The target site can be the cytosol in the case of siRNA, miRNA, mRNA and standard ASO, or the nucleus in case of pDNA, RNA splicing-modulating ASOs, or Cas9/sgRNA.

Apart from stability, the size, shielding, targeting and an efficient endosomal escape, a reduction of carrier-triggered toxicity has to be considered for in vivo nucleic acid delivery. Polycations like PEI have been developed for clinical application with encouraging results, yet the high transfection efficiency goes hand in hand with N/P ratio dependent cytotoxicity. Bio-degradable domains such as the bioreducible ssbb unit, or enzymatically cleavable L-arginine peptides, can be incorporated into the sequence [121,122] to enable the degradation of the oligopeptides into less-toxic small fragments, thereby improving the biocompatibility of nanocarriers.

## 5. Barcoding—A New Mode to Apply Sequences for Finding In Vivo Nanocarriers

Thousands of nanocarrier systems with different structures and properties have been designed and synthesized, with the long-term aim to overcome the pharmacological barriers for in vivo delivery. Due to the laborious nature of in vivo experiments, nanoparticles usually are first evaluated in cell culture systems to select a smaller number of candidates that exhibit the best performance for further in vivo characterization. However, cell culture in vitro experiments may provide information on successful intracellular delivery and bioactivity there, but they cannot predict the efficiency of the whole in vivo delivery process [49,53,184,185]. Therefore, high throughput methods for screening and identification of promising delivery candidates for efficient in vivo delivery are of utmost importance.

A pioneering example of in vivo screening was reported by Seng Cheng and colleagues [186] who analyzed a large library of cationic lipid/pDNA formulations for efficient gene transfer to the mouse lung. By this large screen, a formulation was discovered containing cationic lipid #67, a spermine—cholesterol derivative linked in a T-shape configuration, which was >100-fold more potent in vivo than previous pDNA lipid nanoparticles. A more recent impressive example was published by Dan Anderson and colleagues [187] who generated a lipopeptide library for the formation of siRNA lipopeptide nanoparticles (103 nanomaterials). In vivo screening for hepatocyte gene silencing in mice using a very convenient blood coagulation factor VII assay identified the lead lipopeptide cKK-E12, which subsequently was also found to be highly potent for liver gene silencing in nonhuman primates.

**Figure 6:**
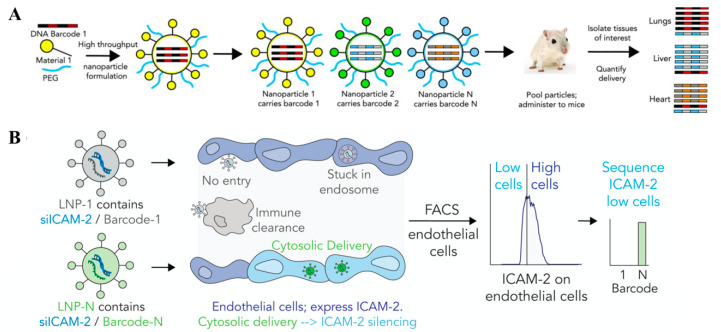
DNA-barcoded nanoparticles for high throughput in vivo carriers. (**A**) **N**anoparticles were formulated to carry a unique DNA barcode and then the LNPs were pooled together and administered simultaneously to mice. Tissues of interest were isolated and delivery was quantified by deep sequencing the barcodes. Reproduced with permission from [188], National Academy of Sciences, 2017. (**B**) “FIND” strategy to quantify functional delivery of LNPs within a single mouse. Nanoparticles were formulated to carry a distinct DNA barcode and siICAM-2 and then the LNPs were pooled together and administered to mice intravenously. After 3 days, ICAM-2^Low^ endothelial cells were isolated and the DNA barcodes within that population were sequenced. (Reproduced with permission from [194], American Chemical Society, 2018).

Using standard in vivo screening for the comparison of tested substances usually requires the use of several animals per substance due to reproducibility reasons. For ethical and economic reasons, a reduction of number of the experimental animals would be preferable. A most innovative solution for this problem was developed by Dahlman and colleagues [188]. They introduced sequence information for optimizing in vivo delivery of nucleic acids in a different mode as discussed in the previous sections. Their work applied “DNA barcoding”, which is a technology previously developed for identification and selection of unique members of libraries such as chemical libraries. For example, researchers had generated DNA-encoded chemical libraries by coupling each of the numerous chemical molecules of a library to a unique nucleic acid sequence (“DNA barcode”) [189]. After high throughput screening of such a library in a functional assay, the identity of the top candidates can be easily revealed by their attached DNA barcodes. Dahlman and colleagues applied such a DNA barcode-based system for screening multiple nucleic acid carriers simultaneously in one single mice and identifying most of the suitable carrier candidates (Figure 6). Chemically distinct lipid nanoparticle (LNP) formulations were prepared by a microfluidics system, where into each nanoformulation a distinct unique DNA barcode oligonucleotide was incorporated. DNA barcodes were based on the Illumina next generation sequencing primer technology and were approximately 60 nucleotide long oligonucleotides, terminally stabilized with phosphorothioates, containing a central variable nucleotide sequence as a barcode and the ends as adapter sequences for subsequent Illumina sequencing. The different nanoformulations were pooled together and injected into a single mouse. The nucleic acid barcodes were recovered at a different time point from different tissues or cells, then Illumina deep sequencing was performed to accurately quantify the distinct barcodes and thus the biodistribution of different nanoparticles [188,190]. Careful dosing studies demonstrated that DNA barcode LNPs can be measured at a low dose, which confirms the feasibility of multiplexing hundreds of nanoparticles in a single experiment [188].

This technology enables one to address new interesting questions on the pharmacology of drug and nucleic acid delivery. For example, LNPs containing cholesterol primarily traffic to liver hepatocytes, which is similar to natural lipoproteins. When comparing the delivery of more than 100 barcoded LNPs containing different cholesterol variants and analyzing distribution into eighteen cell types of the mouse, LNPs formulated with esterified cholesterol were found to be more effective in nucleic acid delivery than LNPs formulated with regular or oxidized cholesterol [191]. LNP containing cholesteryl oleate delivered siRNA and sgRNA more efficiently to liver endothelial cells than to hepatocytes. Information like this can be applied for the rational design of tissue-targeted nanocarriers.

A biodistribution assay named the Quantitative analysis of nucleic acid therapeutics (QUANT) DNA barcoding was developed to compare the difference of nucleic acid nanoparticles delivery to multiple cell types in wild-type and Cav1 knockout mice [192]. QUANT DNA barcodes were rationally designed with a reduced secondary structure to increase DNA polymerase access, which enables digital droplet PCR (ddPCR) readouts and quantifies delivery with very high sensitivity. Direct comparing in vitro and in vivo nucleic acid delivery of 281 barcoded LNPs to endothelial cells and macrophages indicated that in vitro delivery to macrophages and endothelial cells could not predict the delivery to the same cell types in vivo [193].

Beyond monitoring biodistribution only, a novel strategy, named “Fast identification of nanoparticle delivery (FIND)”, was developed by Dahlman’s lab to quantify the functional delivery of hundreds barcoded LNPs to multiple cell types within a single mouse. Functional nucleic acids, such as siRNA, sgRNA and mRNA, were co-formulated into LNPs with a unique DNA barcode. They measured the functional Cre mRNA delivery of more than 250 LNPs to multiple cell types in vivo, and identified two LNPs that efficiently deliver siRNA, sgRNA and Cas9 mRNA to endothelial cells and mediate endothelial cell gene editing [195]. Combined with bioinformatics, the team performed in vivo directed evolution of RNA delivery, and identified one LNPs that effectively delivers siRNA and sgRNA to bone marrow endothelial cells (BMECs) in vivo [194]. Meanwhile, in vivo screening can help to reveal the relations between nanoparticle structures and activities. The tropism of BMEC was not related to the particle size, yet changed with PEG structure and the introduction of cholesterol. For example, the group found a targeting ligand-free LNP containing oxidized cholesterol delivered Cre mRNA into the liver microenvironment 5-fold more than to hepatocytes, suggesting that cholesterol chemical composition played an important role in LNP targeting [196]. By applying a siGFP/DNA-barcoded system in transgenic eGFP mice, the group found that constrained lipid nanoparticles (cLNPs) containing a conformationally constrained adamantane tail can deliver siRNA and sgRNA to splenic T cells in vivo at low doses [197], and in contrast to standard LNPs do not preferentially target hepatocytes. Based on this, the group further demonstrated cLNP containing adamantyl phospholipids can delivery Cre mRNA to liver immune cells at low doses [198]. Like reported before [188], delivery takes place in a size-independent and chemical composition-dependent manner, providing a potential alternative for further active targeting based on chemical modification and not targeting ligands.

By screening hundreds of nanoparticles simultaneously, the high throughput DNA barcode-based system helps the researcher to understand the delivery process of nanocarriers, elucidate chemical composition-activity relations and identify optimized nanoparticles for in vivo gene editing. The DNA barcode sequence may be integrated into nanoparticles without affecting their in vivo behaviors. Up to now the strategy has been successfully applied for LNP formulations, but might be further used in various nanomaterials, including sequence-defined oligopeptides. Combined with rational design, empirical screening, computational prediction and sequencing-generated large datasets, DNA barcode-based systems will accelerate the chemical evolution of nanomaterials for in vivo application.

## 6. Conclusions

The delivery requirements for different nucleic acids differ due to their different physical properties such as sizes, charges and due to different biological requirements such as different intracellular target sites. As nucleic acids are commonly based on anionic phosphodiester backbones, their requirements for systemic administration are similar due to the shared pharmacological barriers. However, non-viral gene delivery system optimized for one type of nucleic acid, e.g., pDNA, cannot directly be applied to another type (e.g., siRNA and PMO). Meanwhile, as discussed in the above sections, moieties such as compaction capacity, stabilization, shielding, endosomal buffering capacity and the transfection efficiency shall be combined and balanced in the optimization of specific nucleic acids delivery. The incorporation of a certain functional unit needs to be balanced with other functional units. PEGylation of polyplexes reduces the unspecific interaction in serum and prolongs the circulation time, yet it also may strongly reduce the transfection efficiency in target cells (known as PEG dilemma). Proper PEG length as well as incorporation with beneficial moieties for enhanced endosomal escape can be adopted to solve this problem. In many cases, the top-ranking nanocarriers in vitro may not perform well in vivo, while optimized nanocarriers that performed well in vivo may not rank in the top in vitro, and would be discarded during in vitro prescreening. Therefore, using the most relevant screening models to select the optimal carriers is vital for further in vivo application.

Benefited from the improved understanding of the natural evolution of viruses and recognition of specific pharmacological barriers, a rational design and synthesis of sequence-defined nanocarriers under optimized chemical conditions might be an effective strategy for optimizing nucleic acids delivery. In contrast to natural carriers, such nanocarriers can profit from a much wider space of artificial amino acids and building blocks. By incorporating bioresponsive domains at a specific site of the sequence, dynamic synthetic artificial “viruses” can enhance the availability of nucleic acid at the required site of action with increased biocompatibility.

Precisely designed oligopeptides by incorporating various functional units in a defined sequence and topology facilitate the establishment of clear structure-activity relations. The relations identified by biophysical characterization and in vitro/in vivo functional screening enable further optimization of the nanocarrier within an evolution-like process. Though gene therapy is still on its rising phase, already 3025 gene therapy clinical trials have been performed worldwide at the current stage (see http://www.abedia.com/wiley). With the development of macromolecular chemistry, supramolecular self-assembly and high throughput screening technique, more and more artificial non-viral nucleic acid products will reach the nanomedicine market in the near future.

## Figures and Tables

**Table 1:** Properties of therapeutic nucleic acids.

Cargo	Properties	Target Site	Function and Mechanism
pDNA	Circular, large (5–15 kbp) double-stranded DNA	Nucleus	Encodes a cDNA expression cassette under the control of a strong promoter/enhancer unit
siRNA	Short noncoding double-stranded RNA with 21–25 bp sequence	Cytosol	RNAi; unwound into single-strand bound in RISC that recognizes the complementary mRNA sequence, resulting in mRNA cleavage.
miRNA	MicroRNA, short noncoding endogenous double-stranded RNA	Cytosol	Regulate gene expression post-transcriptionally by binding of mRNA and thus preventing translation of mRNA into protein
mRNA	Single stranded sequence transcribed from DNA	Cytosol	Translated into proteins in the cytoplasm
sgRNA	Noncoding short RNA sequence binding Cas9 protein	Nucleus	sgRNA guides the nuclease Cas9 to a selective target DNA sequence via complementary binding to make a DSB
ASO	Chemically stabilized short single-stranded antisense oligonucleotide	Cytosol/Nucleus	Bind to mRNA and prevent translation, or to induce exon skipping
PMO	Synthetic uncharged ASO, in which the ribosyl rings and phospodiesters in the backbone are replaced with methylenemorpholine rings and a phosphorodiamidate backbone, respectively.	Nucleus	Bind to pre-mRNA in the nucleus and alter gene splicing, resulting in the exclusion or inclusion of particular genetic fragments in the mature mRNA

pDNA: plasmid DNA; siRNA: small interfering RNA; miRNA: mRNA: messenger RNA; RISC: RNA-induced silencing complexes; sgRNA: single guide RNA; ASOs: antisense oligonucleotides; PMOs: phosphorodiamidate morpholino oligomers; DSB: double-strand break.

**Table 2:** Representative linear and branched HK peptides.

Peptide	Structure	Topology	Functions
**H2K**	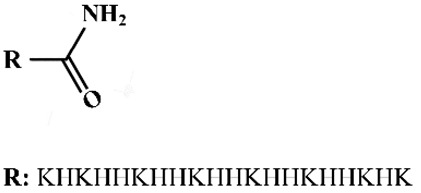	Linear	Ineffective in nucleic acid delivery in vitro, need to combine with cationic carriers as liposomes [45,51]. Far more effective for in vivo pDNA delivery [49].
**H** ^**2**^ **K4b**	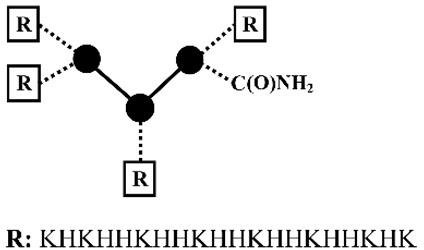	4-branched	Effective in pDNA delivery yet infective for in vitro siRNA transfection as compared with H3K8b [47]. Far more effective for in vivo siRNA delivery [48] as compare with H3K8b.
**H**^**3**^**K4b** H^**3**^**K(+H)4b**	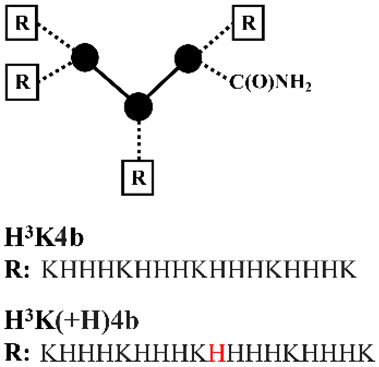	4-branched	H3K4b exhibited better in vitro siRNA transfection than H2K4b [47]. H3K(+H)4b exhibited comparable in vitro siRNA transfection efficiency as compared with H3K8b [53]. 4-branched HK peptide exhibited far more effective gene silencing effect for in vivo siRNA administration [48].
**H** ^**3**^ **K8b**	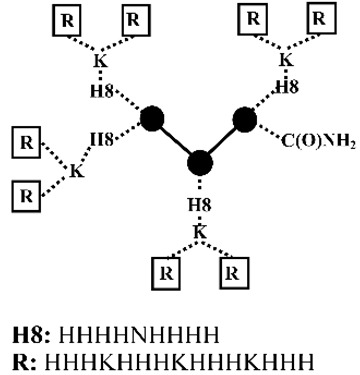	8-branched	Highly effective as carriers for siRNA in vitro transfection as compared with 4 branched H2K4b and H3K4b peptide [47]. Lower in vivo siRNA transfection efficiency as compared with 4 branched H2K4b and H3K4b peptide [48].

Linear H2K peptides with the repeating amino-acid sequence of -HHK-; H2K4b and H2K4bT peptides have four branches with the repeating sequence of -HHK-; highly-branched H3K8b peptides contain histidine-enrich domains and eight terminal branches with the repeating -HHHK- sequence.

**Table 3:** Sequence of cell-penetrating peptides (CPPs) for nucleic acid delivery.

CPPs	Sequences	Cargos
GALA	WEAALAEALAEALAEHLAEALAEALEALAA	DNA [58]
KALA	WEAKLAKALAKALAKHLAKALAKALKACEA	DNA [58]
RALA	WEARLARALARALARHLARALARALRACEA	DNA [58,59], siRNA [60], mRNA [61]
RAWA	RAWARALARALRALARALRALAR	DNA [70]
PepFect3	Stearyl-AGYLLGKINLKALAALAKKIL-NH_2_	SCOs [62], pDNA [63].
PepFect6	Stearyl-AGYLLK(K(K2(tfq4)))INLKALAALAKKIL-NH_2_	siRNA [64]
PepFect14	Stearyl-AGYLLGKLLOOLAAAALOOLL-NH_2_	SCOs [71], pDNA [65], siRNA [72]
NickFect1	Stearyl-AGY(PO3)LLKTNLKALAALAKKIL-NH_2_	SCOs [66], pDNA [63]
NickFect51	(Stearyl-AGYLLG)-δ-OINLKALAALAKKIL-NH_2_	SCOs, pDNA, siRNA [67,68]
NickFect55	(Stearyl-AGYLLG)-δ-OINLKALAALAKAIL-NH_2_	pDNA [69].

(K(K2(tfq4))) is a tetra(trifluoromethylquinoline) derivate.

**Table 4:** Peptide sequence of supramolecular particles for nucleic acid delivery.

Name	Sequences
Glu-KW	Glu-GSGSGS-KKKKKKKK-GGSGGS-WKWEWKWEWKWEWG
C-S_n_-B	C-(GAGAGAGQ)_10_-K_12_
K3C6SPD	KKKC_6_-WLVFFAQQGSPD
H4K5-HC_Bzl_C_Bzl_H	HHHH-KKKK-C12LL-H-C_Bzl_C_Bzl_-H-LLGSPD
PAs	PalA-VVVAAAEEE
P2	TFVETGSGTSKQVAKRVAAEKLLTKFKT
P3	SIRKLEYEIEELRLRIGGG
P4	SIRKLEYEIEELRLRIGGGTFVETGSGTSKQVAKRVAAEKLLTKFKT

PalA: Palmitic acid; Glu: d-glucose.

## Abbreviations

ASGPR	Asialoglycoprotein receptor
ASOs	Antisense oligonucleotides
Cas9	CRISPR-associated protein 9
cLNPs	cLNP constrained lipid nanoparticles
cMBP2	c-Met-binding peptide
CPPs	cell-penetrating peptides
CRC	Cysteine–arginine–cysteine
DBCO	Dibenzocyclooctyne amine
ddPCR	digital droplet PCR
DSB	double-strand break
DTNB	5,5′-Dithio-bis(2-nitrobenzoic acid)
ECO	1-aminoethyl)iminobis[*N*-(oleicylcysteinyl-1-amino-ethyl)propionamide]
EG5	Eglin-5
EHCO	(1-aminoethyl) imino bis [*N*-(oleoyl cysteinyl histinyl-1-aminoethyl) propionamide]
EGFR	Epidermal growth factor receptor
EPR	Enhanced permeation and retention
ESC	Enhanced stabilization chemistry
Fmoc	Fluorenyl methoxycarbonyl
FolA	Folic acid
FR	Folate receptor
FRET	Fluorescence resonance energy transfer
GSH	Glutathione
INF7	glutamic acid-enriched analogue of the influenza hemagglutinin membrane protein HA2
LNPs	Lipid nanoparticles
LPEI	linear PEI
mRNA	Messager RNA
MTX	Methotrexate
NIS	Sodium iodide symporter
NLS	Nuclear localization signal
NPC	Nuclear pore complex
OAAs	Oligoaminoamides
OPSS	ortho-pyridyl disulfide
PAs	Peptide amphiphiles
pDNA	Plasmid DNA
PEG	Polyethylene glycol
PEI	Polyethylenimine
pHPMA	poly(*N*-(2-hydroxypropyl)methacrylamide)
pING4	pDNA encoding inhibitor of growth 4
PMOs	Phosphorodiamidate morpholino oligonucleotides
PT	Pretubulysin
RAFT	Reversible addition-fragmentation chain transfer
RISC	RNA-induced silencing complex
RNAi	RNA interference
RNP	Ribonucleoprotein
SCOs	splice-correcting oligonucleotides
sgRNA	single guide RNA
siRNA	Small interfering RNA
Stp	Succinoyltetraethylene-pentamine
Sph	Succinoyl-pentaethylene hexamine
SPS	Solid phase supported synthesis
SSBB	Disulfide building block = succinoyl-cystamine
tBoc	t-butyloxycarbonyl
TCPs	Targeted combinatorial polyplexes
TLPs	Targeted lipopolyplexes
TNBC	triple-negative breast cancer
Tf	Transferrin
TfR	Transferrin receptor
TTR	Transthyretin
